# *C. elegans* Aging Is Modulated by Hydrogen Sulfide and the sulfhydrylase/cysteine Synthase *cysl-2*

**DOI:** 10.1371/journal.pone.0080135

**Published:** 2013-11-08

**Authors:** Bedoor Qabazard, Samanza Ahmed, Ling Li, Volker M. Arlt, Philip K. Moore, Stephen R. Stürzenbaum

**Affiliations:** 1 School of Biomedical Sciences, King's College London, London, United Kingdom; 2 Neurobiology Group, Life Sciences Institute and Department of Pharmacology, Yong Loo Lin School of Medicine, National University of Singapore, Singapore; University of Louisville, United States of America

## Abstract

Exogenous hydrogen sulfide (H_2_S) administration and endogenous H_2_S metabolism were explored in the nematode *C. elegans*. Chronic treatment with a slow-releasing H_2_S donor, GYY4137, extended median survival by 17-23% and increased tolerance towards oxidative and endoplasmic reticulum (ER) stress. Also, *cysl-2*, a sulfhydrylase/cysteine synthase in *C. elegans*, was transcriptionally upregulated by GYY4137 treatment and the deletion of *cysl-2* resulted in a significant reduction in lifespan which was partially recovered by the supplementation of GYY4137. Likewise, a mammalian cell culture system, GYY4137 was able to protect bovine aortic endothelial cells (BAECs) from oxidative stress and (H_2_O_2_)-induced cell death. Taken together, this provides further support that H_2_S exerts a protective function which is consistent with the longevity dividend theory. Overall, this study underlines the therapeutic potential of a slow-releasing H_2_S donor as regulators of the aging and cellular stress pathways.

## Introduction

Hydrogen sulfide (H_2_S), historically associated with the pungent odour of the naturally occurring gas, has recently attracted extensive attention for its multiple physiological functions and potential contribution to pathological states. In animal cells H_2_S is produced endogenously as a by-product of the transsulfuration (L-cysteine biosynthesis) pathway by the action of two enzymes: cystathionine β-synthase (CBS) and cystathionine γ-lyase (CSE) [1]. Setting aside the potential toxicity mediated via the inhibition of mitochondrial cytochrome *c* oxidase [2] H_2_S has, more recently, been reported to act as an antioxidant with anti-apoptotic, anti-inflammatory and vasodilator activity, as well as a signalling molecule, neuromodulator and cytoprotectant [3]. Indeed, derangement of the H_2_S synthesizing machinery has been linked to inflammation [3], Alzheimer’s disease [4], stroke [5] and diabetes mellitus [6]. 

H_2_S has been reported to regulate cell cycle and survival in healthy cells [7] suggesting it may play a role in cell fate and hence the aging process. Biogerontology and aging research is primarily concerned with unravelling the ambiguity of the aging process and aims to find pharmacological or genetic strategies that control the rate of aging. The “longevity dividend approach” states that any delay of the aging process may also postpone the onset of all aging-related diseases [8]. Given that the monitoring of complete survival profiles in humans is challenging, model organisms such as the nematode *Caenorhabditis elegans* are valuable surrogates, and arguably the premier animal model for aging research. Indeed, previous work reports that the exposure to low concentrations of H_2_S gas result in the extension of the nematodes lifespan [9]. 

However, the *C. elegans* sulphur metabolic pathways have not been characterized in great detail and very little is known about endogenous H_2_S generation in this nematode. Recently, Vozdec and colleagues (2012) [10] proposed that the *ZC373.1* gene encodes the most structurally and functionally similar (though heme-lacking) enzyme to human CBS whereas other paralogs in the same gene family seem to be more structurally related to the plant and bacterial CBS-related enzymes, typically known as sulfhydrylases/cysteine synthases (CYSL). To reflect these structural differences, *ZC373.1* was renamed as *cbs-1* and the other paralogs *cysl* [11]. *K10H10.2*, or *cysl-2*, has been described as a hypoxia inducible factor (HIF) target gene [12] and has been shown to be activated in response to H_2_S and HCN exposure [11] (Budde & Roth, 2011). The same authors proposed that CYSL-2 can generate H_2_S in *C. elegans* by catalyzing the first step reaction between cysteine and HCN in the HCN assimilation pathway resulting in the production of β-cyanoalanine and H_2_S [11]. 

Sulfide salts such as sodium hydrosulfide (NaHS) and sodium sulfide (Na_2_S) have been widely used to release H_2_S but they do so instantaneously which does not align well with the slow release patterns for this gas observed *in vivo* [13]. In contrast, the water soluble H_2_S donor morpholin-4-ium 4-methoxyphenyl(morpholino)phosphinodithioate (GYY4137, first synthesised by Professor C-H Tan, Department of Chemistry, National University of Singapore, and now commercially available from Sigma Aldrich) is characterized by a slow and sustainable H_2_S release that more closely mimics physiological levels [14]. GYY4137 is active *in vitro* and *in vivo* [14] and protects against lipopolysaccharide (LPS)-induced inflammation [13,14].

This study aims to unravel the biological response to H_2_S using the genetically tractable model organism *C. elegans*. We explored the involvement of exogenous H_2_S (via the slow-releasing donor GYY4137) as a modulator of the aging process and protection against environmental stress in the nematode *C. elegans*. Furthermore, we characterized the phenotypic changes associated with knocking out the gene *K10H10.2* (*cysl-2*) which was hypothesized to be implicated in endogenous H_2_S fate/response. Key results were validated in cell culture experiments utilizing bovine aortic endothelial cells (BAECs) to model biological processes in the context of a higher eukaryotic system with a vascular system which notably is absent in the nematode. 

## Materials and Methods

### Chemicals, *C. elegans* handling procedures and strains

The slow-releasing H_2_S donor morpholin-4-ium 4-methoxyphenyl(morpholino) phosphinodithioate, or GYY4137, was freshly prepared as an aqueous solution (final concentration of 100 µM). To maximize contact time with the gas (H_2_S), staged (freshly hatched and age-synchronous) L1 larvae were first rotated in liquid medium (2 parts OP 50, grown in LB broth, and 1 part M9 buffer) at room temperature (22-23°C) for 48 hours in a sealed tube (Note: to guard from hypoxia-induced effects, liquid exposures were performed in 15ml tubes with an air to liquid ratio of 2:1. In addition, after 24h, the worms were pelleted by gentle centrifugation (2500g, 2 minutes) and new exposure medium (with fresh drugs) added). Following the liquid exposure, worms (typically young L4 larvae) were plated on solid nematode growth medium (NGM) inoculated with the same concentration of GYY4137; drug exposure was continued on plates for the entire experiment. Strains used in this study were Bristol N2 (wild type); *cysl-2* (*ok3516*); *daf-16*(*mgDf50*); *sir-2.1(ok434*), *skn-1* (*zu67*); and *jnk-1*(*gk7*). Nematodes were maintained at 20°C on Nematode Growth Media (NGM) seeded with a bacterial lawn of *Escherichia coli* strain OP50 as a food source. Synchronization of worm cultures was achieved by hypochlorite treatment of gravid hermaphrodite. In all experiments the respective negative controls were run in parallel. 

### Life-cycle assessment (brood size, growth and lifespan)

All demographic assays were conducted at 20°C. Brood size was determined by placing single L4 nematodes into each well of a 12-well plate and transferred daily to new plates until the termination of the reproductive period. Hatched offspring (per worm) were counted on the second day after transfer and plotted as the average cumulative number of eggs produced. Growth was assessed by observing the changes in nematode body size (flat surface area and length) throughout all developmental stages from first larval to adulthood at 24 hours time interval over a 10 day period. Briefly, digital photographs of at least 15 nematodes per treatment condition were taken at the same magnification using a digital camera attached to a microscope (Nikon, UK) and quantified using the Image-Pro® Express v5.1 software (Media Cybernetic, Wokingham, UK). For life span assays, following the 48-hour period of liquid exposure, L4 larvae were placed on seeded NGM plates and transferred daily to new plates during the reproductive period. When reproduction ceased, worms were transferred to fresh plates every day and death was verified by failure to move or respond to gentle prodding with a platinum wire. Worms that crawled off the plates or died due to vulval bursting were censored. All lifespan assays were repeated at least twice.

### Analysis of intracellular oxidative free radicals

Intracellular hydrogen peroxide (H_2_O_2_)-related reactive oxygen species (ROS) were measured in *C. elegans* using 2,7-dichlorodihydrofluorescein diacetate (H_2_DCF-DA; Sigma-Aldrich, UK) as described previously [15]. Age-synchronized L4 stage worms treated in liquid medium with or without GYY4137 (100 µM final concentration) for 48 hours were collected into 100 µl of 0.1% PBST (Phosphate Buffered Saline with 1% Tween 20) in 1.5 ml microcentrifuge tubes. Each sample consisted of 1000 worms per group and 3 replicates per treatment. The worms were homogenized with a sonicator (Branson Sonifier 250, VWR Scientific) to break the outer cuticle and 50 µl of sample combined with 50 µl of 100 µM H_2_DCF-DA in PBST dye mixture into a 96-well plate and incubated at 37°C in the dark. Fluorescence measurements were taken every 30 minutes for 3 hours on a FLx800 Microplate Fluorescence Reader (Bio-Tek Synergy HT Instruments, Winooski, VT USA; excitation at 485 nm and emission at 528 nm). 

### Stress resistance assays

Worms were treated and handled as mentioned above, however at day 6 (post-hatch) the worms were transferred to plates containing either H_2_O_2_ (0.8 mM) which according to [16,17] induces death, cadmium (100 µM), or tunicamycin (10 µg/ml). After 48 hours, survival was assessed as described above. To assess thermal tolerance, control and GYY4137-treated adult worms were incubated at 37°C (7 hours) then scored for viability (essentially as described by [18]).

### Polymerase Chain Reaction (PCR)

After backcrossing (four times) the *cysl-2* (*ok3516*) strain with the wild type N2, single worm genomic PCR was conducted to confirm the presence of a homozygous *cysl-2* (*ok3516*) deletion. Quantitative real-time PCR was performed using the ABI Prism7000 platform (Applied BioSystems, Warrington, UK) to calculate the relative fold change in gene expression using the comparative 2^-ΔΔCT^ method, normalizing to *rla-1*, an invariant housekeeping gene [19,20]. Total RNA was extracted from L4 worms with Tri-reagent (Sigma, UK), and cDNA was produced by oligo(dT) priming. 

### Measurement of H_2_S synthesizing activity

Enzymatic H_2_S synthesis in worm tissue homogenates was measured as described previously [21] with the following modifications: 430 µl supernatant derived from approximately 4000 age-synchronous L4 worms raised using standard plate (NGM) culturing conditions (numbers were defined by titres and normalized to equal worm count) and the homogenate was incubated with 20 µl pyridoxal 5’-phosphate (PLP, 2 mM) and the substrate L-cysteine (20 µl, 10 mM) and sealed with a double parafilm layer to avoid leakage of H_2_S gas generated after incubating the tube in a 37°C water bath for 45 minutes. Baseline controls contained trichloroacetic acid (TCA; 10% w/v, 250 µl) prepared in parallel to obtain the basal H_2_S background level. At the end of the incubation period, zinc acetate (1% w/v, 250 µl) was injected to trap the H_2_S followed by TCA (10% w/v, 250 µl) to terminate the reaction. Subsequently, *N*,*N*-dimethyl-*p*-phenylenediamine sulfate dye (NNDPD; 20 µM, 133 µl) in 7.2 M HCl was added, followed by the addition of FeCl_3_ (30 µM, 133 µl) in 1.2 M HCl. After centrifugation (14,000 rpm for 4 min at 4°C), absorbance (670 nm) of the resulting methylene blue in the supernatant was measured using a 96-well microplate reader (Tecan Systems Inc., CA, USA) and compared against a standard curve of NaHS (concentrations ranging from 3.125 to 250 µM). All 3 biological samples were assayed in duplicate and results expressed as nmol H_2_S formed per mg protein per min. Protein level in each homogenate was estimated using the Bradford assay (Bio-Rad, CA, USA). 

### Cell culture and viability tests

Bovine aortic endothelial cells (BAECs; isolated from discarded aortae sourced, with permission, from the licensed abattoir ABP Guildford, UK) were obtained as a gift from the Nandi lab, King’s College London. Cells were isolated from aortae using 0.5 mg/ml collagenase (Boehringer) and cultured in 75 cm^2^ flasks in phenol-free low-glucose Dulbecco’s modified Eagle’s Medium (DMEM) supplemented with 10% fetal calf serum (FCS), 5.5 mM D-glucose, 5 mM L-glutamine, penicillin/streptomycin (100 U/ml) and maintained at 37°C under a humidified atmosphere of 5% CO_2_ and 95% air and used in passage 3-10. Cells (seeded at 1.2×10^4^/well into 96-well plates) were grown for 24 hours, after which a fresh medium supplemented with the required drug treatments (GYY4137, or the pro-oxidant hydrogen peroxide (H_2_O_2_) was added to the wells. After 24 hour incubation with the drugs, cell viability was measured employing the CellTiter-Blue (CTB) Cell Viability Assay (Promega, UK Ltd) according to the manufacturer’s instructions. 

### Statistics and data analysis

All survival curves were plotted by the Kaplan-Meier method and median survival values were compared by log-rank (Mantel-Cox) testing with 95% significance limits using the GraphPad Prism v5 software. All other assays are presented as mean ± standard error of the mean (SEM) for 3 biological replicate samples. ANOVA’s followed by *post hoc* testing or Student’s *t*-tests were applied as deemed appropriate using GraphPad Prism v5 statistical software and significant differences were identified where *p* <0.05.

## Results

### Evidence for enzymatic H_2_S generation in *C. elegans* in vitro

The true concentration of H_2_S in *C. elegans* cells under our experimental conditions remains unknown as techniques for the accurate intracellular measurement of H_2_S in nematodes have yet to be developed. However, using the zinc trapping method, we observed that worm lysates generated considerable amounts of H_2_S, approximating 1.4 ± 0.06 nmol H_2_S/mg protein/min.

### The slow-releasing H_2_S donor GYY4137 attenuates intracellular ROS generation and improves survival and growth in wild type *C. elegans* nematodes

**Figure 1:**
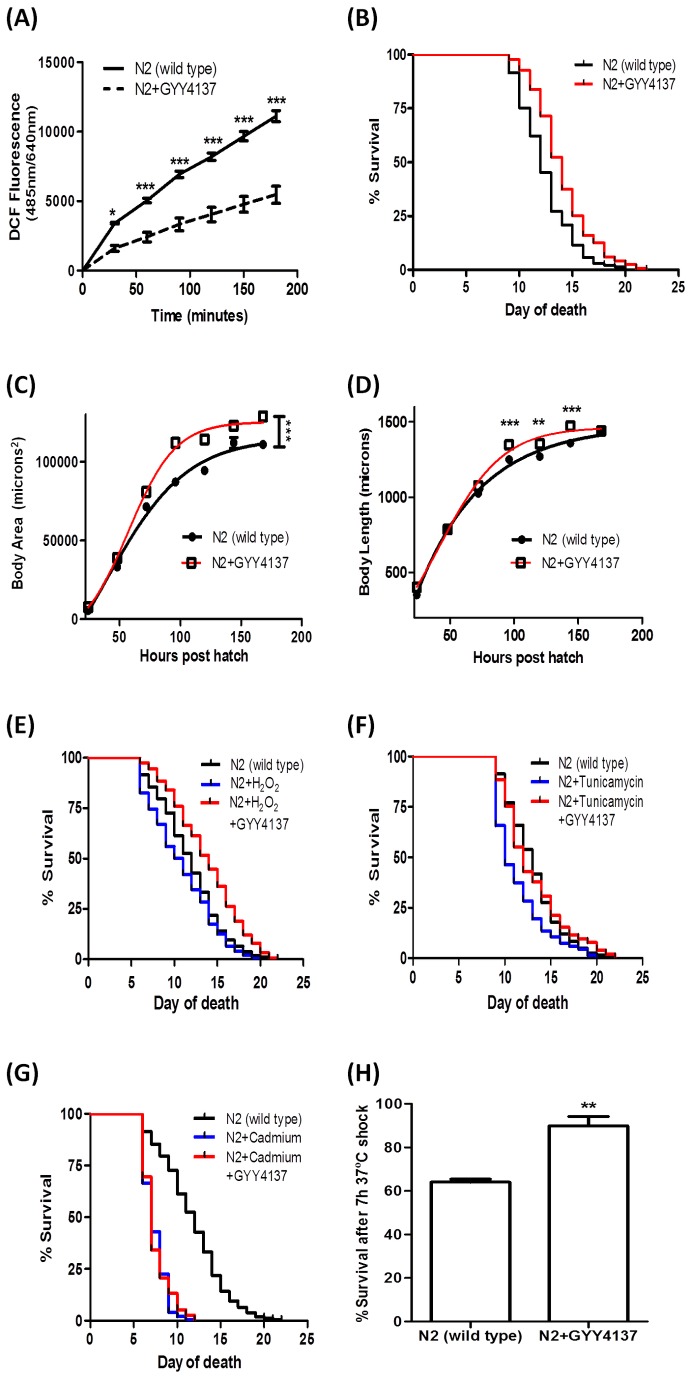
The slow-releasing H_2_S donor GYY4137 mediates an aging and stress response in *C. elegans*. (A)Intracellular levels of ROS were obtained from age-synchronized worms assayed at L4 stage following treatment with vehicle (control) or GYY4137 (100 µM) for 48 hours in liquid medium. Each bar represents data from 3 independent experiments with at least 2000 worms in each treatment group and normalized by the calculated protein content (* and ***, statistically significant c.f. control; p < 0.05 and p < 0.001, respectively). GYY4137 treatment improved (B) survival and (C-D) growth (body area and length) in wild type worms. Nematodes were exposed to the drug from L1 larval stage until death (day of death refers to the age (days) post L1); survival curves were plotted by the Kaplan-Meier method and median survival values were compared by log-rank (Mantel-Cox) test with 95% significance using GraphPad Prism v5 software. GYY4137 treatment improves the resistance of wild type nematodes to (E) oxidative, (F) ER and (H) thermal stressors, but notably not to (G) cadmium-induced stress.

GYY4137 did not affect the growth characteristics of the bacteria suggesting that the concentrations used did not induce a drug-bacteria interaction (data not shown). According to the free radical theory of aging, accumulation of oxidative damage is a leading cause for aging and degenerative disease. To assess changes in intracellular ROS levels, we quantified, by means of the H_2_DCF-DA assay [15], H_2_O_2_ concentrations in control and GYY4137-treated nematodes. A remarkable attenuation of the basal H_2_O_2_ level was observed in GYY4137-treated worms compared to untreated controls (Figure 1A), suggesting a novel antioxidative mechanism of GYY4137. GYY4137 treatment induced a significant two to four days extension of the median lifespan of the wild type N2 nematodes in multiple independent trials (log-rank, *p* <0.0001, Figure 1B and Table 1). *C. elegans* growth was assessed from the L1 larval stage for seven consecutive days. Whilst the liquid pre-exposure in the absence of GYY4137 had little effect on the overall growth curve in wild type worms (data not shown), GYY4137 treatment induced a significant increase in body area and length from the third day onwards (Figure 1C and D). 

**Table 1:** Lifespan summary.

Condition	Trials	Mean Lifespan ±SEM (days)		Median Lifespan (days)		Log-rank test	Control/Treated (n)
		-GYY4137	+GYY4137	-GYY4137	+GYY4137		
N2 Normal Condition (no stress)	Trial 1	13.5 ± 0.3	15.7 ± 0.33	13	15	< 0.0001	160/196
	Trial 2	13.1 ± 0.4	16.8 ± 0.3	13	16	< 0.0001	130/200
	Trial 3	11.6 ± 0.5	14.6 ± 0.4	12	16	< 0.0001	50/118
	Trial 4	12.1 ± 0.02	13.7 ± 0.2	12	14	<0.0001	163/204
N2 Oxidative stress (H_2_O_2_ 0.8 mM)	Trial 1 -H_2_O_2_	12.3 ± 0.4	-	12	-	0.0256^a^	60
	Trial 1 +H_2_O_2_	10.8 ± 0.5	12.6 ± 0.5	10.5	12	0.0111^b^	60/60
	Trial 2 -H_2_O_2_	12.1 ± 0.4	-	12	-	0.0170^a^	61
	Trial 2 +H_2_O_2_	10.6 ± 0.5	14.4 ± 0.6	10.5	14	< 0.0001^b^	60/60
	Trial 3 -H_2_O_2_	12.6 ± 0.4	-	12	-	0.0346^a^	80
	Trial 3 +H_2_O_2_	11.0 ± 0.5	13.9 ± 0.6	11	13	0.0002^b^	40/40
	Trial 4 -H_2_O_2_	12.0 ± 0.3	-	12	-	0.0188^a^	114
	Trial 4 +H_2_O_2_	10.6 ± 0.6	14.2 ± 0.6	10	13	0.0022^b^	40/40
N2 ER stress (Tunicamycin 10 µg/ml)	Trial 1 -Tunic	14.3 ± 0.4	-	14	-	0.0352^a^	44
	Trial 1 +Tunic	12.0 ± 0.5	14.5 ± 0.6	11	14	0.0296^b^	23/23
	Trial 2 -Tunic	13.5 ± 0.4	-	14	-	0.0489^a^	51
	Trial 2 +Tunic	12.2 ± 0.6	13.6 ± 0.5	11	13	0.0434^b^	23/23
	Trial 3 -Tunic	14.6 ± 0.4	-	14	-	0.0157^a^	66
	Trial 3 +Tunic	12.2 ± 0.5	14.6 ± 0.6	12	14	0.0128^b^	21/23
N2 Metal stress (Cadmium 100 µM)	Trial 1 -Cd	12.3 ± 0.4	-	12	-	< 0.0001^a^	60
	Trial 1 +Cd	7.3 ± 0.2	7.1 ± 0.2	7	7	0.6725^b^	60/60
	Trial 2 -Cd	12.1 ± 0.4	-	12	-	< 0.0001^a^	60
	Trial 2 +Cd	7.5 ± 0.2	7.7 ± 0.2	7	7	0.4097^b^	60/60
	Trial 3 -Cd	12.0 ± 0.4	-	12	-	< 0.0001^a^	80
	Trial 3 +Cd	7.6 ± 0.2	7.3 ± 0.2	7	7	0.4598^b^	40/40
	Trial 4 -Cd	12.0 ± 0.4	-	12	-	< 0.0001^a^	114
	Trial 4 +Cd	7.4 ± 0.2	7.1 ± 0.2	7.5	7	0.1141^b^	40/40
*cysl-2 (ok3516)*	Trial 1 N2	14.6 ± 0.3	-	14	-	< 0.0001^c^	95
	Trial 1 *cysl-2*	10.9 ± 0.3	12.9 ± 0.2	11	12	0.0254^d^	55/107
	Trial 2 N2	14.0 ± 0.3	-	13	-	< 0.0001^c^	87
	Trial 2 *cysl-2*	11.2 ± 0.2	12.8 ± 0.2	11	12	0.0050^d^	89/104

^a^ compared to control (-GYY4137) without stressor. ^b^ compared to control (- GYY4137) with stressor. ^c^ compared to N2 wild type. ^d^ compared to control *cysl-2*. n, number of animals tested.

### GYY4137 treatment protects the wild type nematodes against oxidative, ER, and thermal stressors but not cadmium-induced metallic stress

Since longevity and resistance to various forms of stress are correlated, it is conceivable that the observed beneficial effects of GYY4137 on *C. elegans* aging may be via an effect on cellular stress resistance pathways. This possibility was tested by investigating the response of nematodes to several independent stress inducers, namely H_2_O_2_ (oxidative stress), tunicamycin (ER stress), cadmium (metal-induced stress) and 37°C heat shock (thermal stress). Exposing the control nematodes to the oxidative stressor H_2_O_2_ resulted in a marked drop in survival, an effect that could be partially rescued by GYY4137 treatment (Figure 1E and Table 1). This indicates that GYY4137 can also enhance survival during oxidative stress. To examine whether GYY4137 can also induce an anti-ER stress response, worms were exposed to tunicamycin (10 µg/ml) and scored daily for survival. The addition of GYY4137 effectively induced longevity in tunicamycin-challenged nematodes (Figure 1F and Table 1). Because heavy metals are well-known redox-active stress-inducers, an underlying cause of aging and degeneration [22], we investigated whether GYY4137 can confer resistance to the heavy metal, cadmium. Although cadmium was very effective in reducing the lifespan of worms, no difference in death rate was observed between GYY4137-treated and untreated animals (Figure 1G and Table 1). Failure of GYY4137 to protect against cadmium toxicosis suggests that GYY4137 is not a universal stress-buffer but targets specific physiological pathways/endpoints. Heat resistance is often linked to enhanced survival in *C. elegans* [18]. We observed that GYY4137-treated worms were more thermotolerant than untreated controls, where on average 90% of GYY4137-treated animals but only 64% of untreated controls were viable following a 7 hour heat shock at 37°C (Figure 1H and Table 1). This finding suggests that GYY4137 triggers physiological changes that are manifested by an improved survival at high temperature. 

### GYY4137 regulates the mRNA expression of aging-associated and stress-related genes in wild type *C. elegans*

**Figure 2:**
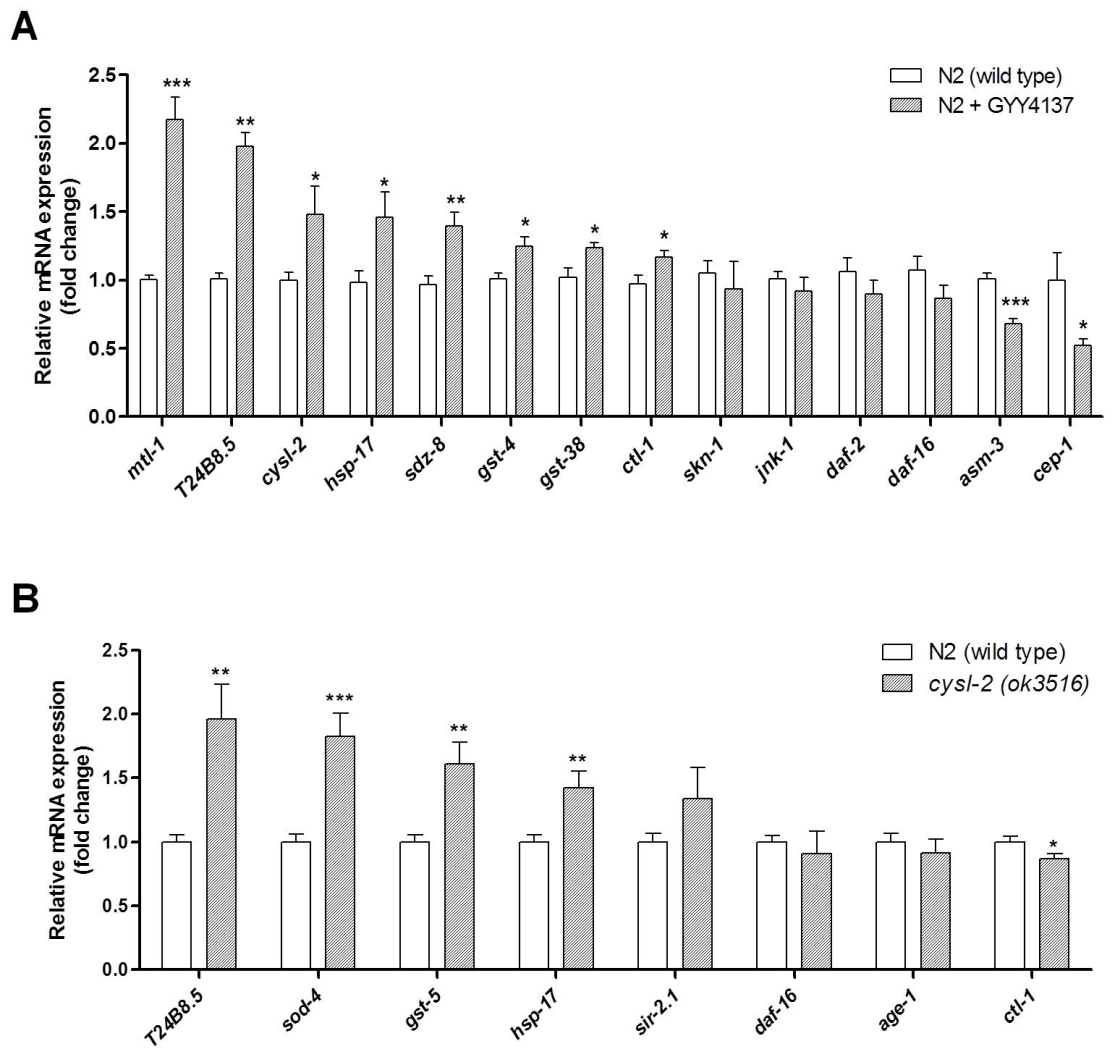
The transcriptional response of wildtype exposed to GYY4137 and in *cycl-2* mutants. Genes involved in aging- and stress-related pathways were selected to evaluate, by qPCR, expression changes associated with GYY4137 treatment (A) in wild type *C. elegans* in the absence (-) or presence (+) of GYY4137 (100 µM) and (B) in *cysl-2* mutants compared to N2 wild type (in the absence of GYY4137).

**Figure 3:**
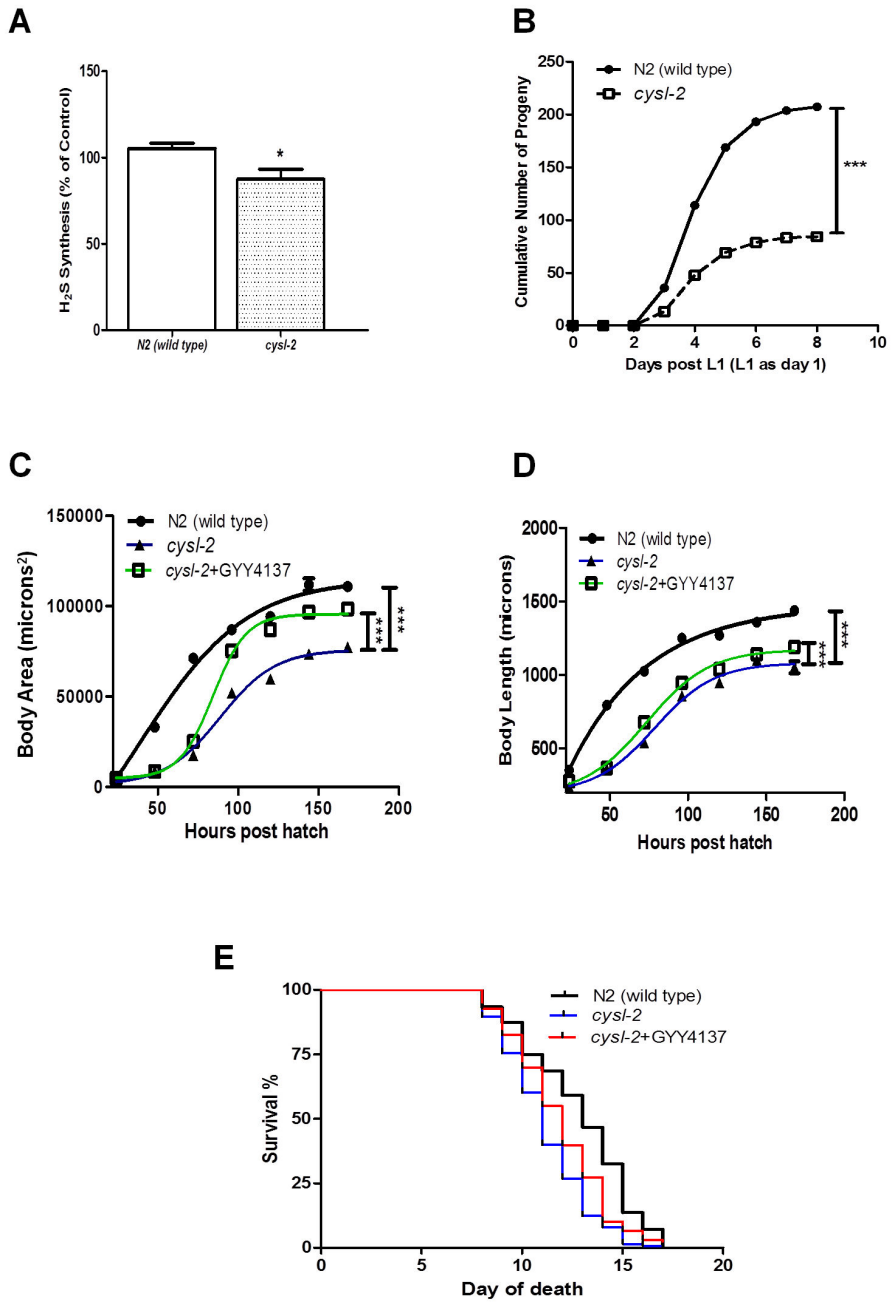
The *cysl-2* mutant phenotype. (A) The *cysl-2* mutant has a reduced rate of enzymatic H_2_S synthesis from the added substrates L-cysteine. (B) The *cysl-2* mutant is marked by a decline in reproductive output compared to wild type worms. (C) Body area and (D) length are significantly lower in *cysl-2* compared to age-matched wild type worms, and GYY4137 treatment (100 µM) effectively rescued this phenotype. (E) Lifespan is reduced in *cysl-2* compared to wild type (median survival of 11 days vs. 12 days, respectively) and the addition of GYY4137 (100 µM) reverses this effect and increases the median survival of *cysl-2*.

Quantitative real-time PCR was performed to investigate whether GYY4137 could affect the expression of genes widely accepted to play a role in aging and stress resistance (Table S1, Figure 2A). GYY4137 did not significantly modulate the expression of *daf-2*, *daf-16*, *skn-1* and *jnk-1*; in contrast, a modest but statistically robust transcriptional induction was observed for *gst-4*, *gst-38*, *ctl-1*, *mtl-1*, *sdz-8*, *cysl-2* and suppression of *cep-1* and *asm-3*. Given that *cysl-2* was upregulated by GYY4137 treatment, it was deemed to be a suitable target for further investigation. The expression of a selection of ageing and stress-related genes was examined by qPCR in the *cysl-2* mutant and compared to wild type (Figure 2B). No significant difference (compared to wild type) was observed in *daf-16*, *sir-2.1* and *age-1* expression. However, the oxidative stress genes (*gst-5, sod-4* and *hsp-17*) and the SKN-1 regulated gene *T24B8.5* were statistically more abundant in the *cysl-2* mutant, which may indicate an adaptive response to an internal stress or disturbance of the physiological state caused by *cysl-2* gene deletion (Figure 3B).

### The *cysl-2* mutant is characterized by a reduced endogenous H_2_S synthesis, decreased reproductive capacity, growth retardation and reduced lifespan; effects which could be ameliorated by GYY4137 treatment

Detailed phenotype analysis of *cysl-2*(*ok3516*), a mutant nematode lacking a predicted CBS-related (CYSL) enzyme, revealed characteristic phenotypes. The *cysl-2* mutant generated, compared to wild type worms, 13% less H_2_S from the added substrate L-cysteine (Figure 3A). Moreover, the total cumulative number of viable offspring was significantly lower in *cysl-2* mutants (84±0.2) compared to wild type worms (207±0.5), suggesting that reproduction is severely impaired (Figure 3B). In addition, *cysl-2* worms were only half the size of age-matched wild type animals (Figure 3C and D). A very distinctive feature of *cysl-2* mutants was the occurrence of premature death which on average was some 2 days earlier than the wild type nematodes (Figure 3E).

GYY4137 treatment of *cysl-2* reversed the aging phenotype resulting in a one-day enhancement of median lifespan (12 days in GYY4137-treated vs. 11 days in untreated *cysl-2* mutants, p <0.01, Log-rank, Figure 3E and Table 1). In addition, growth parameters (body area and length) showed significant improvement by GYY4137 treatment (Figure 3C and D). 

### Exploring the effects of exogenous H_2_S on BAEC viability

**Figure 4:**
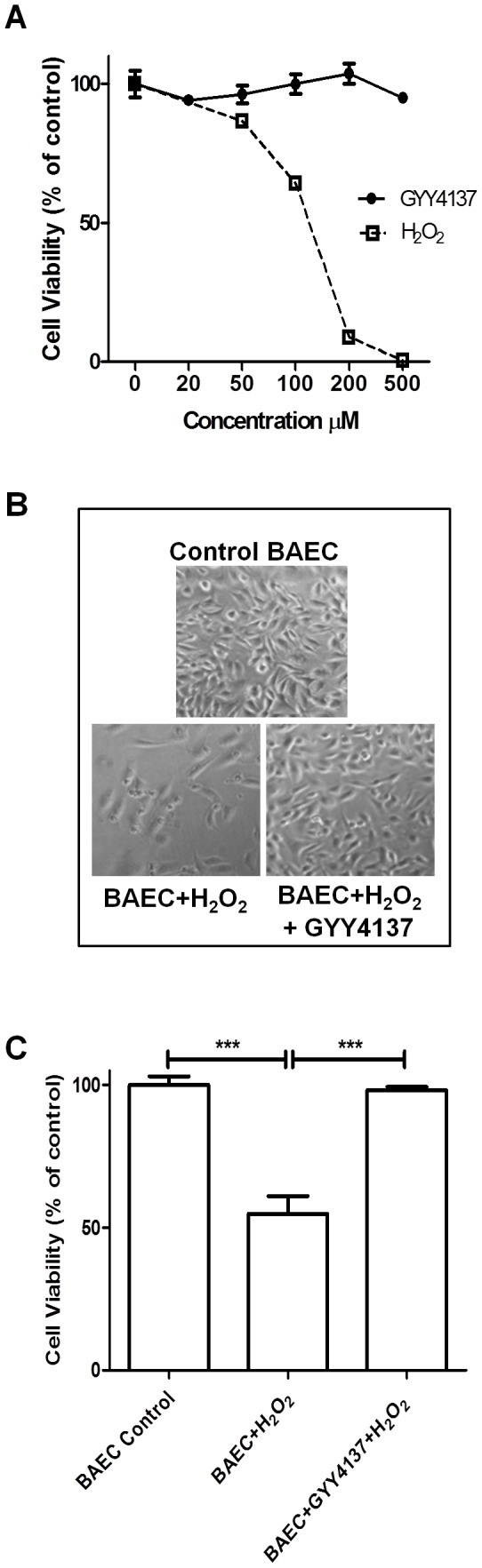
GYY4137 protects BAECs from oxidative stress (H_2_O_2_)-induced cell death. (A) Cell viability was determined with the CTB assay. Cell viability testing after 24 hours incubation of BAEC cultures with a range of concentrations of the slow-releasing H_2_S donor GYY4137 or the oxidative stress inducer H_2_O_2_. (B) Representative images of BAEC treated cultures in the presence or absence of H_2_O_2_ (100 µM) and GYY4137 (100 µM). (C) Effect of GYY4137 on cell death induced by H_2_O_2_ (100 µM). BAEC were exposed to H_2_O_2_ in the presence or absence of GYY4137 (100 µM) for 24 hours and cell viability was determined by means of the CTB assay. Data are expressed as percentage of untreated control from 6 repeats.

Whilst GYY4137 did not induce cytotoxic effects over the dose range tested, a finding which is in agreement with previous reports [14,23,24], H_2_O_2_ treatment provoked a concentration-dependent cell lethality (Figure 4A). To validate the hypothesis that GYY4137 processes protective antioxidant action against H_2_O_2_, GYY4137-treated and untreated BAEC were exposed to H_2_O_2_ (100 µM) for 24 hours. The CTB assay confirmed that pre-treatment with GYY4137 improves cell viability significantly, providing further support that GYY4137 prevents H_2_O_2_-induced oxidative death (Figure 4B and C). 

## Discussion

### Exogenous H_2_S affects survival, growth and intrinsic stress resistance

Many long-lived *C. elegans* mutants are resistant to oxidative stress [25], heat [18], UV radiation [26] as well as heavy metals [22]. It should be noted here that although median lifespan measurement is a sensitive endpoint, *C. elegans* is very sensitive to the differences in growth conditions and even wild type can vary between laboratories from 11.8 days to 20 days [27]. Therefore relative changes in lifespan can be made when using the same strain and conditions but cross-laboratory comparisons are potentially misleading. The median lifespan of the worms used here was at the lower end of the acceptable norm which was possibly due to the fact that worms were transferred daily, (rather than during the reproductive period) to maintain a constant slow release of H_2_S, or indeed due to the modified liquid/plate exposure regime. Nevertheless, the results aligns well with the hypothesis that stress resistance and longevity are interconnected as exemplified by the ability of GYY4137 to increase the lifespan of worms subjected to oxidative, ER and thermal stressors. This putative antioxidant or radical scavenging activity (via the reduction in ROS generation) agrees with recent findings that demonstrate the protective effect of GYY4137 against oxidative stress-induced cell death in chondrocytes [24]. H_2_S is a strong reducing agent and scavenges free radicals in mammalian cells, which could be a mechanism through which longevity and stress tolerance are generated by GYY4137. However, GYY4137 treatment did not protect worms against cadmium-induced toxicity. Others have shown that NaHS is able to protect Alfalfa seedling roots against cadmium-induced oxidative damage, however only in the presence of nitric oxide (NO) [28] which may explain this discrepancy.

### GYY4137 induced longevity and stress resistance: insights into the mechanism of action

H_2_S is lipophilic and can readily access all cellular compartments without the need for a receptor or specialist membrane transport carrier. It is therefore perhaps not surprising that H_2_S has been shown to trigger complex responses in experimental model systems. Given that H_2_S is a strong reducing agent with antioxidant properties, one prominent target is likely to be the cellular redox balance, which may have a direct or indirect effect on the observed life-cycle phenotypes. An initial investigation to explore the molecular pathways mediating GYY4137 pro-longevity action was conducted using knock-out strains for the genes *daf-16*, *sir-2.1*, *skn-1* and *jnk-1*. Unlike wild type N2, which coped well with the initial liquid based GYY4137 exposure, *skn-1* and in particular *jnk-1* arrested during this first 48 hour period and growth resumed after being plated onto solid media. This 2-day growth lag will likely have affected the median lifespan values. However taken together these results tentatively suggest that lifespan extension by GYY4137 is less pronounced in *daf-16*, *sir-2.1* and *jnk-1* (Figure S1). Given that *skn-1* was shown to be essential for survival when subjected to the pure H_2_S gas [29], we were surprised to note that the lifespan of the *skn-1* mutant subjected to GYY4137 was only reduced by a (statistically insignificant) day. The observed difference may be explained either due to the use of different experimental platforms and downstream data processing or because the different route and duration of administration and the subsequent change in the rate and speed of drug exposure between the pure gas and the releasing pro-drug GYY4137.

To further explore the molecular basis of GYY4137 action, quantitative real-time PCR was performed. Although *skn-1* and *jnk-1* genes were not directly induced by GYY4137, some of the downstream genes in these pathways were affected. The putative SKN-1 transcriptional target *gst-4*, which encodes a glutathione S- transferase-4 involved in the phase II oxidative stress response, was induced statistically significant, though modest in amplitude (1.2-fold). A similarly non-dramatic induction was reported by Miller et al. 2011 [29] who observed a 2.2-fold induction of *gst-4* expression following a 24 hour exposure to 50 ppm H_2_S gas. SKN-1 Dependent Zygotic transcript family member, *sdz-8*, and *cysl-2* which are under the transcriptional control of SKN-1 were found to be significantly induced by GYY4137, thus reflecting an augmented SKN-1 activity that increased the expression of the antioxidant targets of SKN-1. Surprisingly, *cysl-2*, is also under the transcriptional control of SKN-1 which suggests a possible causative link between CYSL-2, its product (H_2_S) and the cellular stress response and longevity via SKN-1 signalling. Interestingly, our results showed an induction of *T24B8.5* (ShK-like toxin gene) which is one of the SKN-1 regulated genes and SKN-1/H_2_S modulated genes identified by others [29,30]. The significant induction of T24B8.5 following GYY4137 exposure observed in our study and after H_2_S gas exposure in Miller et al. 2011 [29] suggests that *T24B8.5* may be a novel H_2_S-regulated gene or is at least crucial for the response to H_2_S gas or the releasing compounds. 

Nevertheless, certain DAF-16 targets were also induced, including metallothionein *mtl-1* (also shown to be upregulated in [29]), a catalase (*ctl-1*) and a heat shock protein (*hsp-17*), thus pointing towards a complex pattern of exogenous H_2_S signalling which involves the dual activation of SKN-1 and DAF-16 target genes known to be protective against environmental stress and contribute to longevity. Moreover, the PMK-1/MAPK signalling pathway seems to determine the response to H_2_S as shown by the lack of lifespan extension in *jnk-1* mutants (Figure S1). On the other hand, the qPCR showed a reduced expression of the *C. elegans* p53 ortholog *cep-1*, the expression of which is known to be inversely related to lifespan [31]. Li et al. 2008 [14] observed no pro-apoptotic effect or p53 induction in mouse fibroblasts upon GYY4137 treatment. Another gene suppressed by GYY4137 was *asm-3*, which encodes a putative acidic sphingomyelinase (ASM). Mammalian ASM is involved in converting sphingomyelin to ceramide which in turn acts as a secondary messenger during the cellular stress response [32]. Accumulation of ceramide underlies several diseases and injuries induced by environmental stress; therefore current research is ongoing to find novel inhibitors of ASM to protect against stress [33]. The finding that GYY4137 can reduce ASM expression is clearly of interest and opens a novel mechanism for exogenous H_2_S as a protective agent that boosts the internal defence mechanisms against stress and thus promotes a healthy and extended life. 

### The effect of endogenous H_2_S on *C. elegans*

One of the key enzymes responsible for endogenous H_2_S synthesis in mammals is cystathionine ß-synthase (CBS), the rate-limiting enzyme in the transsulfuration pathway [34-36]. It was demonstrated that in *Drosophila* the inhibition of CBS caused a 30-46% reduction in H_2_S levels [37], whereas adenovirus-mediated overexpression of CBS was associated with higher H_2_S levels in mice [38]. Bioinformatic analysis of the *C. elegans* databases (www.wormbase.org) identified several orthologs of the CBS-related family, namely *K10H10.2* (*cysl-2*), *ZC373.1*(*cbs-1*), *F54A3.4* (*cbs-2*) *C17G1.7*(*cysl-1*) and *R08E5.2*(*cysl-3*). CYSL-1 catalyzes the formation of cysteine and acetate from *O*-acetylserine and H_2_S, whereas CYSL-2 catalyzes the generation of β-cyanoalanine and H_2_S from cysteine and HCN [11]. The knock-out mutant strain *cysl-2*(*ok3516*) used in this study was characterized by an impaired H_2_S synthesis capacity as demonstrated, *in vitro*, by the addition of cysteine, which supports the notion of the CYSL-2-catalyzed production of H_2_S from cysteine and HCN interaction proposed by Budde and Roth (2011) [11]. Therefore *cysl-2*(*ok3516*) is a promising model to explore the effects of endogenous H_2_S deficiency in *C. elegans* even if the observed decline in H_2_S level was modest, which may be due to the presence of other gene orthologs and a resultant compensatory H_2_S release. In addition, reproductive output, body size, and survival were compromised in *cysl-2* deletion mutant, indicating that CYSL-2 is essential for normal development and reproduction [39-43] and that the loss of this enzyme with the concomitant disturbance of the internal H_2_S generation leads to an accelerated aging phenotype in nematodes. However, although the mutant allele was backcrossed four times, it should be noted that, though unlikely, it is possible that the observed phenotypes are due to residual background mutations. It therefore would be prudent to validate the observed effects either by RNAi gene silencing or by generating a second (independent) loss-of-function allele. Nevertheless, RNAi of another *C. elegans* CBS homolog, *ZC373.1*, was also associated with defects in development and egg laying [10]. In vertebrates, severe growth retardation and early death were observed in CBS^-/-^ KO mice [44], and RNAi-mediated knockdown of *Drosophila* CBS blocked the lifespan extension associated with dietary restriction, whereas CBS overexpression markedly increased *Drosophila* longevity [37]. Furthermore, H_2_S levels and CBS enzyme activity were shown to be reduced in homocystinuria [45,46] and liver cirrhosis [47]. Collectively, our findings are in agreement with these reports and suggest a vital role of endogenous H_2_S and CBS-related enzymes in maintaining normal development and healthy lifespan. 

### Validation of results in Bovine Aortic Endothelial Cells (BAEC)

Ageing is one of the major risk factors in the development of age-related cardiovascular diseases such as hypertension, coronary heart disease and atherosclerosis. Substantial evidence for the involvement of H_2_S in the pathogenesis of hypertension and atherosclerosis has been provided (reviewed in 1). Despite the well-documented hypotensive, anti-inflammatory and antioxidant effects of H_2_S *in vivo* and *in vitro*, its precise role in endothelial cells homeostasis is not clear. Endothelial dysfunction, collectively caused by oxidative stress, inflammation, cell adhesion and cell proliferation, is a major underlying mechanism in the pathogenesis of atherosclerosis [48]. Preservation of the endothelium integrity and function is mandatory for protection against atherosclerosis and cardiovascular pathologies. To date, few studies have addressed the contribution of H_2_S to the defense against oxidative damage in endothelial cells. Hence, we investigated the protective effects of exogenous H_2_S in endothelial cells *in vitro* in response to H_2_O_2_-induced oxidative stress. To test the hypothesis that H_2_S is implicated in improving survival and protecting the vascular endothelium against cellular injury in the present study, we used BAECs as a mammalian model system and GYY4137 as a source of H_2_S. GYY4137 did not provoke a concentration-dependent cytotoxicity which is consistent with previous reports ruling out any cytotoxicity for GYY4137 in several cell lines, namely rat aortic vascular smooth muscle cells [14], hepatic HepG2 cells in mice [23], human non-cancer WI-38 and IMR90 fibroblasts [49] and in mesenchymal progenitor cells (MPC) and chondrocytes [24]. In contrast, the fast-releasing sulfide salts Na_2_S and NaHS have been shown to illicit a toxic effect or induce death in a range of cell lines in some studies [50-54]. This may be a direct consequence of the slow H_2_S release rate of GYY4137 mimicking the physiological release pattern compared to the acute instantaneous release from NaHS and Na_2_S that can be harmful. Whilst GYY4137 did not induce cytotoxic effects over the dose range tested in our study, a finding which is in agreement with previous reports [14,23,49], H_2_O_2_ treatment provoked concentration-dependent cell lethality. This support the results from our *C. elegans* study, namely that H_2_S protects against oxidative stress injury as evidenced by the markedly improved cell survival following GYY4137 pre-treatment compared to H_2_O_2_ treatment alone. 

These finding are in agreement with the reported protective antioxidant role of H_2_S in neurons [55], vascular smooth muscle cells [56], myocytes [57] and endothelium [58]. It is conceivable that the mechanism underlying the protective effect of H_2_S in BAECs is a result of a strong reducing or direct scavenging action. Furthermore, H_2_S released from Na_2_S [59] and garlic oil [60] has been shown to upregulate endogenous antioxidants via Nrf2 signaling pathway. This is further supported by our qPCR results in *C. elegans* showing a dependency of GYY4137 action on SKN-1, the worm ortholog of Nrf2, and an induction of multiple antioxidant genes such as glutathione S-transferase, SOD and catalase. However, further studies are needed to define protein expression and enzyme activity of these antioxidant enzymes in BAECs following GYY4137 treatment to define the precise antioxidant role of H_2_S in these cells. 

In conclusion, the present work demonstrates that the disturbance of the endogenous H_2_S system in *C. elegans* is detrimental for survival, reproduction and growth. Exogenously applied H_2_S promotes growth and enhances the survival profile. The protective effect of exogenous H_2_S might be explained in the context of antioxidation and ROS scavenging activation directly and indirectly via stimulating SKN-1, JNK-1 and/or downstream targets. An analogous protection was observed in bovine endothelial cells. These findings provide tantalizing insights into the role of endogenous H_2_S in aging and cellular response to stress and highlight the potential of exogenous H_2_S donors as pharmacological interventions to delay the aging process and protect against age-associated diseases. 

## Supporting Information

Figure S1 **GYY4137-induced longevity is less pronounced in *jnk-1, skn-1anddaf-16* and reversed in sir*-*2.1.** See Table S2 for details.
(TIF)

Table S1 **Functional annotation of transcripts measured in Figure 2 (information adapted from www.wormbase.org**).
(DOCX)

Table S2 **GYY4137 mechanism of action investigation using mutant strains (lifespan summary).**
(DOCX)
