# Supplementary figures and images for: C. elegans Aging Is Modulated by Hydrogen Sulfide and the sulfhydrylase/cysteine Synthase cysl-2
Source: PLoS One. 2013 Nov 8;8(11):e80135. doi: 10.1371/journal.pone.0080135 (PMC3832670; doi:10.1371/journal.pone.0080135)

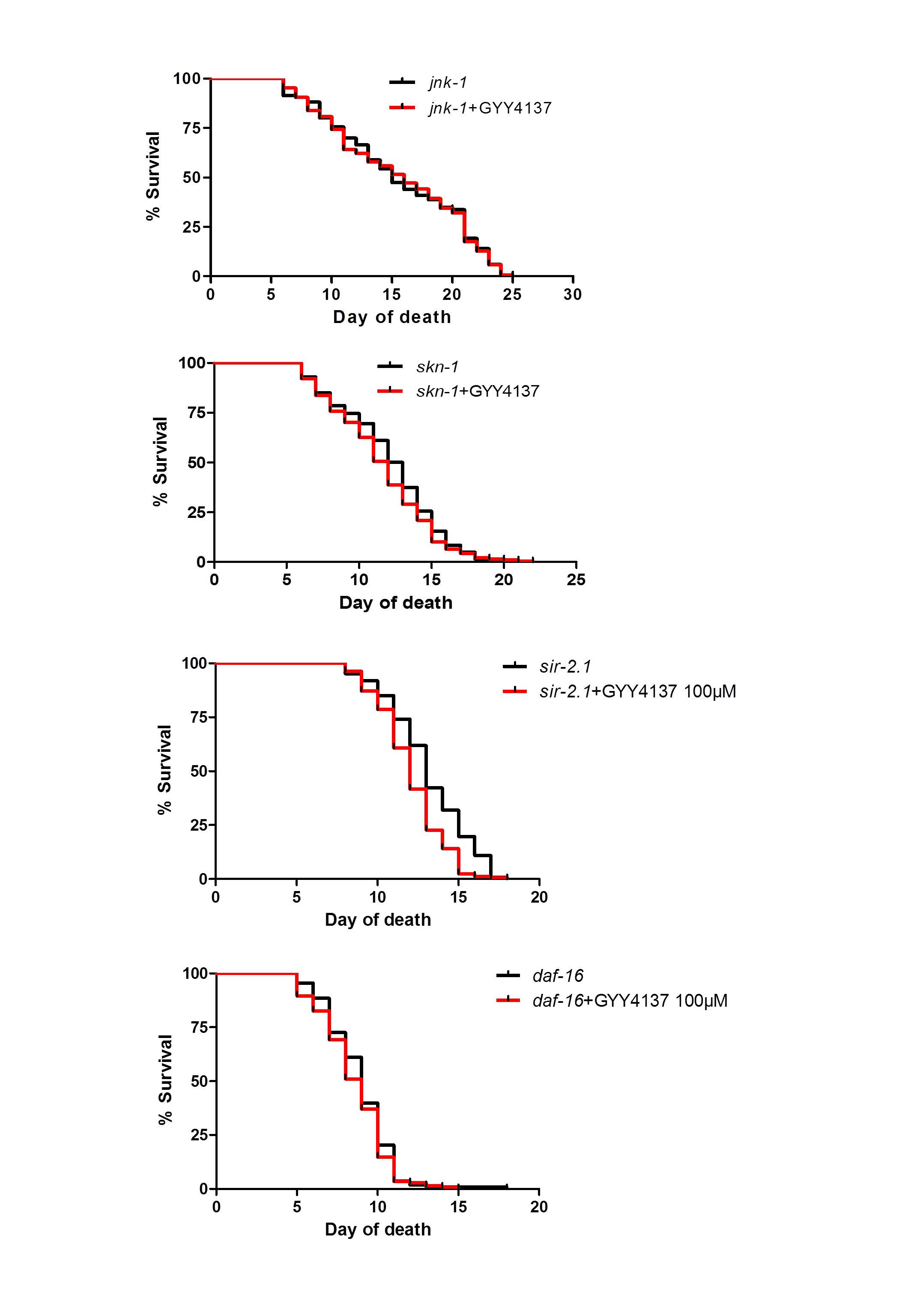

Supplement: Figure S1 — GYY4137-induced longevity is less pronounced in jnk-1, skn-1anddaf-16 and reversed in sir-2.1. See Table S2 for details. (TIF) [file pone.0080135.s001.tif]
