# Supplementary material for: C. elegans Aging Is Modulated by Hydrogen Sulfide and the sulfhydrylase/cysteine Synthase cysl-2
Source: PLoS One. 2013 Nov 8;8(11):e80135. doi: 10.1371/journal.pone.0080135 (PMC3832670; doi:10.1371/journal.pone.0080135)
Supplement: Table S1 — Functional annotation of transcripts measured in Figure 2 (information adapted from www.wormbase.org). (DOCX) [file pone.0080135.s002.docx]

**Table S1: Functional annotation of transcripts measured in Figure 2 (information adapted from www/wormbase.org)**

| Gene | Function |
| --- | --- |
| *mtl-1* | Encodes small, cysteine-rich, metal-binding protein; functions in metal detoxification and homeostasis and in stress adaptation. |
| *T24B8.5* | Encodes an ShK-like toxin peptide; expression is regulated by PMK-1/p38 MAPK signaling pathway and the ATF-7 transcription factor. |
| *cysl-2* | Encodes a homolog of sulfhydrylases/cysteine synthases. |
| *hsp-17* | Encodes a heat shock protein predicted to function as a molecular chaperone that protects cells from heat-induced protein aggregation and denaturation. |
| *sdz-8* | Dependent Zygotic transcript. |
| *gst-4* | Encodes a putative glutathione-requiring prostaglandin D synthase; *gst-4* accumulation increases in response to paraquat. |
| *gst-38* | Encodes a glutathione-S-transferase; predicted to function as a phase II detoxification enzyme and its expression is altered in response to stress. |
| *ctl-1* | Encodes a catalase; likely antioxidant enzyme to protect cells from reactive oxygen species; contributes to extended lifespan seen in *daf-2* mutant animals. |
| *skn-1* | Encodes a bZip transcription factor orthologous to the mammalian Nrf) transcription factors; functions in the *p38* MAPK pathway to regulate the oxidative stress response and in parallel to DAF-16/FOXO in the DAF-2-mediated insulin/IGF-1-like signaling pathway to regulate adult lifespan. |
| *jnk-1* | Encodes a serine/threonine kinase; required for normal coordinated locomotion as well as for normal adult lifespan and response to heat and oxidative stress. |
| *daf-2* | Encodes a receptor tyrosine kinase, an insulin/IGF receptor ortholog; DAF-2 is required for processes including adult longevity, reproduction, stress resistance. |
| *daf-16* | Encodes forkhead box O (FOXO) homologue; transcription factor acts in the insulin/IGF-1-mediated signaling (IIS) pathway to regulates dauer formation, longevity, fat metabolism, stress response, and innate immunity. |
| *asm-3* | Acid SphingoMyelinase. |
| *cep-1* | Encodes an ortholog of the human tumor suppressor *p53*, promotes DNA damage-induced apoptosis, required for normal meiotic segregation in the germ line, and affects sensitivity to hypoxia-induced lethality and longevity in response to starvation. |
| *sod-4* | Encodes an extracellular Cu2+/Zn2+ superoxide dismutase (SOD); required for redox regulation of e.g. axon pathfinding in the PVQ interneurons, insulin/IGF-1 signaling, and vulval development. |
| *gst-5* | Encodes a glutathione-S-transferase that inhibits CEP-1- and HUS-1-dependent germline apoptosis. |
| *sir-2.1* | Yeast SIR related. |
| *age-1* | Encodes ortholog of the phosphoinositide 3-kinase (PI3K)*p110* catalytic subunit; central component of the insulin-like signaling pathway required for regulation of metabolism, life span, dauer formation, stress resistance, salt chemotaxis learning, fertility, and embryonic development. |
