# Supplementary material for: C. elegans Aging Is Modulated by Hydrogen Sulfide and the sulfhydrylase/cysteine Synthase cysl-2
Source: PLoS One. 2013 Nov 8;8(11):e80135. doi: 10.1371/journal.pone.0080135 (PMC3832670; doi:10.1371/journal.pone.0080135)
Supplement: Table S2 — GYY4137 mechanism of action investigation using mutant strains (lifespan summary). (DOCX) [file pone.0080135.s003.docx]

**Table S2: GYY4137 mechanism of action investigation using mutant strains (lifespan summary)**

|  | Mean Lifespan ±SEM (days) | | Median Lifespan (days) | |  |  |  |
| --- | --- | --- | --- | --- | --- | --- | --- |
| Strain | -GYY4137 | +GYY4137 | -GYY4137 | +GYY4137 | Log-rank test | Control/Treated (n) |  |
| *daf-16 (mgDf50)* | 8.88 ± 0.19 | 8.47 ± 0.18 | 9 | 9 | 0.2512 | 113/144 |  |
| *sir-2.1 (ok434)* | 13.14 ± 0.20 | 12.06 ± 0.16 | 13 | 12 | < 0.0001 | 176/166 |  |
| *skn-1 (zu67)* | 12.69 ± 0.24 | 11.46 ± 0.24 | 13 | 12 | 0.1452 | 213/190 |  |
| *jnk-1 (gk7)* | 14.63 ± 0.39 | 14.81 ± 0.38 | 15 | 16 | 0.7704 | 230/230 |  |

GYY4137 concentration 100 µM. Survival curves were plotted from the pooled data of 3 independent experiments. Statistical significance of the difference between the survival curves was determined by log-rank test using the Kaplan-Meier survival analysis (GraphPad Prism v5). n, number of animals tested.
